# When Attention-Deficit/Hyperactivity Disorder reaches adulthood

**DOI:** 10.1192/j.eurpsy.2022.2273

**Published:** 2022-09-01

**Authors:** A.S. Morais, R. Gomes, N. Descalço

**Affiliations:** Hospital Garcia de Orta, EPE, Serviço De Psiquiatria E Saúde Mental, Almada, Portugal

**Keywords:** adult psychiatry, adhd

## Abstract

**Introduction:**

Attention-Deficit/Hyperactivity Disorder (ADHD) was classically considered a childhood-onset neurodevelopmental condition. Over the past 40 years, it became evident that it can persist during adulthood.

**Objectives:**

The purpose of the authors is to describe the characteristics of ADHD in adults and the specific comorbidities, proposing an approach to these patients.

**Methods:**

A brief non-systematized review is presented, using the literature available on PubMed and Google Scholar.

**Results:**

Only 40-50% of children and adolescents with ADHD will have symptoms that persist into adulthood (estimated adult prevalence of 2.8% across 20 countries; 25% in prisons). A more subtle presentation in adults and the difficulty to access past medical history, lead to diagnosis and treatment rates of lower than 20% (versus 50% in children). Well-characterized core symptoms in children evolve into a predominance of inattention symptoms. They became adults with marked disorganization, difficulties in completing tasks and managing time. Emotional dysregulation is a very prevalent symptom in this population. The comorbidities rate increase over time (reaching 75% of patients).

**Conclusions:**

Adults (or even older subjects) with cognitive and/or behavioural complaints should be submitted to systematic screening for ADHD. Non-treated ADHD symptoms in adulthood are associated with severe impairment, therefore adjustments in the health care system to support the transition from child to adult services are needed.

**Disclosure:**

No significant relationships.

